# Effectiveness of Warm-Up Exercises with Tissue Flossing in Increasing Muscle Strength

**DOI:** 10.3390/jcm11206054

**Published:** 2022-10-13

**Authors:** Anna Hadamus, Tomasz Jankowski, Karolina Wiaderna, Aneta Bugalska, Wojciech Marszałek, Michalina Błażkiewicz, Dariusz Białoszewski

**Affiliations:** 1 Department of Rehabilitation, Faculty of Dental Medicine, Medical University of Warsaw, 02-091 Warsaw, Poland; 2 Students Scientific Society for Physiotherapy, Department of Rehabilitation, Faculty of Dental Medicne, Medical University of Warsaw, 02-091 Warsaw, Poland; 3 “Fizjopunkt Orlik” Rehabilitation Clinic, 04-041 Warsaw, Poland; 4 Institute of Sport Sciences, The Jerzy Kukuczka Academy of Physical Education, 40-065 Katowice, Poland; 5 Faculty of Rehabilitation, The Józef Piłsudski University of Physical Education in Warsaw, 00-809 Warsaw, Poland

**Keywords:** tissue flossing, floss band, vascular occlusion, muscle strength, muscle endurance, warm-up

## Abstract

Tissue flossing is an increasingly popular method in physiotherapy and sports. There is a belief that tissue flossing can improve range of motion and muscle strength, shorten muscle recovery time, and reduce the risk of injury. The aim of this study was to analyse the effectiveness of tissue flossing for immediately improving muscle strength in recreational athletes when it is performed during warm-up. All participants were randomly assigned to either an experimental group (*n* = 36) or a control group (*n* = 34) using a random number generator. The experimental group (*n* = 36) performed an intervention comprising exercises with muscle tissue flossing and exercises without flossing. The control group (*n* = 30) performed the same protocol without a floss band. Muscle strength was measured for knee flexion end extension at three speeds (60, 120, and 180 °/s) 3 times. Analysed parameters include peak torque, work, and power related to body weight, flexors–extensors ratio, and time to peak torque. There were no significant changes in the muscle strength parameters from before to after the warm-up in either group (*p* > 0.05). Significantly lower values of peak torque, work, and power were observed in the experimental group during the warm-up with the floss band applied to muscles (*p* < 0.05). No clinically significant changes in time to peak torque or flexors–extensors ratio were observed. A single application of flossing does not improve muscle strength or power and can even reduce individuals’ maximum muscle strength capabilities.

## 1. Introduction

The idea of enhancing strength training via the restriction (occlusion) of blood flow dates back to the mid-1960s in Japan, where it is known as KAATSU. The KAATSU method was developed by Yoshiaki Sato. The occlusive effect in KAATSU training is achieved with narrow elastic tourniquets placed around limbs near joints. Elsewhere, occlusion training refers to blood flow restriction training (BFRT) or low-load blood flow restriction (LL-BFR). For the BFRT technique, wider bands or pressure cuffs are tightened manually or pneumatically so that the inflow of arterial blood is not blocked but venous outflow from the area is blocked [1]. Ongoing and published studies have shown that BFRT is useful in strength training, indicating that this method may stimulate muscle hypertrophy to the same level as high-load resistance training and may be an effective means for increasing muscle strength and muscle mass, even in highly trained individuals. Numerous publications have demonstrated the effectiveness of BFR resistance training at loads of less than 50% of a single maximum repetition, although the underlying processes have yet to be fully explained [2,3,4,5,6]. Despite these numerous reports of the effectiveness of this type of training, negative effects have also been indicated. Adverse effects have included blood clot formation, muscle cell damage, and abnormal blood pressure exercise responses or neural responses (such as numbness in the leg or arm). In light of these adverse effects, caution is advised for individuals with cardiovascular disease in particular [6,7].

Along with tourniquets and cuffs, floss bands are also used for exerting external pressure on tissues. Flossing has been popularised as VooDoo Flossing by Starrett and Cordoza [8]. They pointed out numerous advantages of flossing-mediated pressure, including improvements in joint mobilisation, joint range of motion, the mobility of connective tissue, and the quality of muscle contractions and pain relief. Once the brace removal occurs, there is an abrupt increase in perfusion to the joint and tissues, aiding in the renewal of damaged muscle and joint structures [9]. Elastic floss bands may also reduce joint or muscle oedema; compression forces compel the excess fluid into the lymphatic system, which then helps the body excrete the fluid, which may be useful in relieving delayed-onset muscle soreness (DOMS) [8,10]. However, some studies have suggested that floss bands do not reduce DOMS [11].

Tissue flossing is considered to be a method that is moderately effective in increasing patients’ range of motion [12] and can be applied by the patient independently without continuous support from a physiotherapist. Flossing has also been shown to be useful for athletes, for example, during warm-up, to rapidly increase a joint’s mobility before performing activities requiring maximum effort, such as those in training or competitions [1,13]. On the other hand, our previous study has shown no advantage in comparison to the warm-up procedure without a floss band in increasing trunk flexion measured by sit and reach test [14]. Moreover, only a few studies have assessed the effect of soft tissue flossing on muscle strength parameters, which are very important for performance during training.

There are several factors, that increase the risk of injury, including non-modifiable factors like age, anatomical knee structure, congenital ligaments laxity, and previous injuries, as well as modifiable factors like environment, technique, body weight, muscle laxity, and muscle strength [15,16,17,18]. The relationship between injury rate and muscle strength is visible, especially in the knee joint, which transfers high forces and is stabilized both by ligaments and muscles. Insufficiency of the knee flexors and extensor strength can cause dynamic knee valgus being one of the main knee injury risk factors [15,19,20]. Other researchers showed a relationship between muscle strength and the hamstring strain injury risk [16], meniscus damage progression [21], or re-injury following ACL reconstruction [22,23]. Muscle activation exercises are performed during warm-up to increase the possibility to generate high muscle strength during the main exercise sessions or competitions, and therefore to reduce the risk of injury [24,25]. Some other supporting techniques, like tissue flossing, can be applied additionally to enhance the warm-up effects. There are only a few studies concerning the influence of tissue flossing on muscle strength [13,26,27,28], but the results are inconclusive. The present study investigated the strength parameters of thigh muscles, as they determine lower limb function in many sports and can be a factor in predicting the risk of injury [16,29,30].

The aim of the study was to assess the effect of flossing during the pre-exercise warm-up on selected strength parameters of the knee flexors and extensors. Based on previous studies, it was hypothesized an increase in muscle strength, work and power parameters. Moreover, the time to achieve peak torque was expected to shorten.

## 2. Materials and Methods

### 2.1. Participants

The study enrolled 70 recreational athletes (47 women and 23 men) aged 18–29 years, participating in regular physical exercise sessions 3–4 times per week, min. 30 min each. All participants were randomly assigned to either an experimental group (*N* = 36) or a control group (*N* = 34) using the random number generator in Microsoft Excel. An interview to determine whether individuals met the following exclusion criteria: current musculoskeletal complaints, a history of lower limb surgery or injury within the preceding year, cardiac, vascular, or respiratory disorders, blood coagulation disorders, pregnancy, and cancer (at present or within the five years before the study). All participants were informed about the potential risks associated with the tests, especially the tissue flossing procedure, and agreed to participate in the study.

Four participants from the control group did not complete the experiment due to musculoskeletal complaints that occurred during the isokinetic measurement. Therefore, the control group included finally 30 athletes. The characteristics of the participants that completed the study protocol are in Table 1.

### 2.2. Ethical Approval

The study protocol was approved by the Bioethics Committee of the Medical University of Warsaw (no. KB/217/2020). The work was carried out in accordance with the Declaration of Helsinki.

### 2.3. Measurements

Leg dominance was defined using the revised version of the Waterloo Footedness Questionnaire (WFQ-R) [31]. Muscle strength in the dominant leg only was then measured with the Humac Norm system (CSMi Inc., Stoughton, MA, USA) under isokinetic conditions for knee flexion and extension (concentric/concentric) in an open kinetic chain at the following speeds, following relevant standards [32]: 60 degrees per second (5 repetitions), 120 degrees per second (7 repetitions) and 180 degrees per second (10 repetitions). Before each series, the participants performed two trial repetitions followed by a 5-s break. Between each series, the participants rested for 20 s.

The following parameters were measured separately for the flexors (Flx) and extensors (Ext): peak torque (PT) [Nm], work per repetition (WR) [Nm], mean power (MP) [W], and time to peak torque (TPT) [s]. All torque values included gravity correction. Peak torque, work per repetition and mean power values are then related to body weight (BW) in kilograms. For each parameter flexors-extensor ratio was calculated as follows:

$$Rat_{FlxExt}=\frac{x_{Flx}}{x_{Ext}},\;$$

where $x_{Flx}$ is the value of the parameter for flexors, and $x_{Ext}$ is the value of the parameter for extensors.

### 2.4. Procedures

At the beginning, each participant was tested with WFQ-R. Then, participants performed an 8 min initial warm-up, consisting of slow running (300 m), high knee skips (300 m), 10 squats, skipping for 30 s, and a cycloergometer for 2 min with ca. 30% resistance. All participants were familiar with performed exercises.

#### 2.4.1. Experimental Group

**Figure 1:**
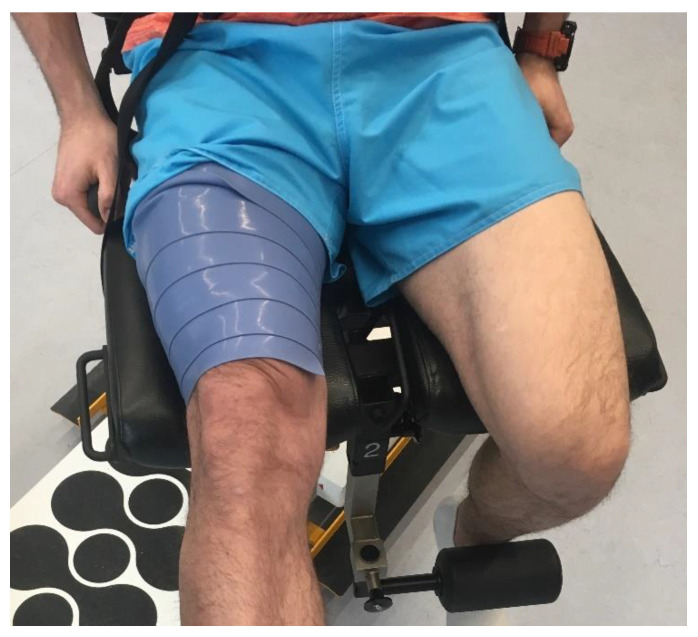
Application of a floss band on tight muscles.

After initial warm-up the experimental group performed the first isokinetic measurement, followed by a 1 min break. Then, a plum (strong) Flossband (Sanctband, WAGUS GmbH, Germany) was applied to the participant’s muscles during this time. The band was always applied by the same physiotherapist (T.J.) who was highly experienced in performing this technique. The band was applied to the tight muscles of the dominant leg, starting from the epicondyles level along the course of the tight muscles proximally while maintaining 50% tension and with 50% overlap of the previous part of the band (Figure 1) [33,34,35]. This procedure lasted about 1 min. Immediately after, a participant performed the second isokinetic measurement, which was followed by a 30-s break. The floss band was removed during this time. The warm-up was continued then for the next 5 min, including medium-speed running (200 m), a cycloergometer for 2 min with ca. 60% resistance, and a trampoline jumps for 1 min. Then, the third isokinetic measurement was performed.

#### 2.4.2. Control Group

Participants assigned to the control group started after initial warm-up with the first isokinetic measurement, followed by a 2 min break. Immediately after, a participant performed the second isokinetic measurement, which was followed by a 30-s break. The warm-up was continued then for the next 5 min, including medium-speed running (200 m), a cycloergometer for 2 min with ca. 60% resistance, and a trampoline jumps for 1 min. Then, the third isokinetic measurement was performed. The same time intervals were kept in the control group as in the experimental group.

### 2.5. Statistical Analysis

Statistical analysis was performed using PQStat 2021 software v. 1.8.2.238 (PQStat Software, Poznań, Poland). Shapiro–Wilk test showed that the variables have non-normal distribution. Because of this and small group sizes, non-parametric test (Friedmann’s ANOVA with post hoc Dunn–Bonferroni test) was used to analyse differences between three measurements (1—before floss band application; 2—with floss band applied on muscles in the experimental group, 3—at the end of a warm-up) within the groups. The results were considered statistically significant for *p* < 0.05.

The effect size was estimated using Cohen’s d. Cohen’s d was counted for combinations between measurements 1, 2 and 3, as follows:

$$d=\frac{m_{a}-m_{b}}{SDpool};\;\;\;\;\;SDpool=\sqrt{\frac{SD_{a}^{2}+SD_{b}^{2}}{2}}$$

where: m_a_, m_a_—means from measurement a and b, respectively; SD_a_ and SD_b_—standard deviations from measurements a and b, respectively (a, b = 1, 2, 3 and a ≠ b). The ranges of effect size for Cohen’s d was as follows: d ≤ 0.5—small, 0.5 < d ≤ 0.8—medium; d > 0.8—large [36].

## 3. Results

### 3.1. Peak Torque per Body Weight

**Figure 2:**
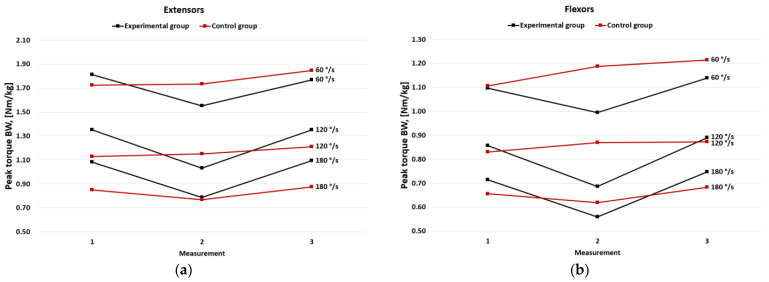
Mean of peak torque per body weight in the experimental and the control group for (**a**) extensors and (**b**) flexors.

In the experimental group, PT per BW values both in extensors and flexors were significantly lower in the second measurement at all three speeds (*p* < 0.05), but they returned to the baseline in the third measurement. Flexors’ peak torque per BW was slightly higher in the third measurement. In the control group, no significant changes were observed between all three measurements at all speeds, but a detailed analysis of the values showed a slight increase in generated PT per BW, both for extensors and flexors (Figure 2).

### 3.2. Work per Repetition per Body Weight

**Figure 3:**
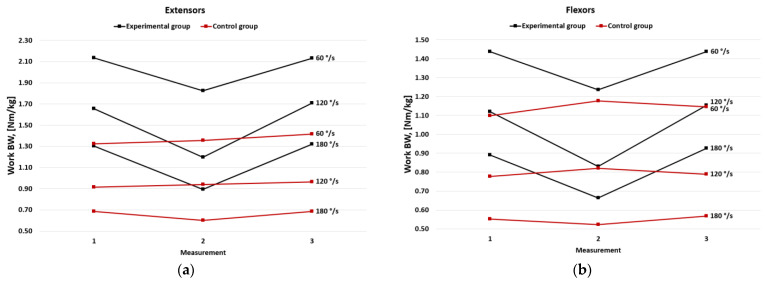
Mean of work per repetition per body weight in the experimental and the control group for (**a**) extensors and (**b**) flexors.

WR values related to BW decreased significantly in the second measurement and then increased in the third measurement (*p* < 0.05) in the experimental group. There were no significant differences between measurements no. 1 and 3. No statistically significant differences were observed in the control group, although a slight increase was observed (Figure 3).

### 3.3. Mean Power per Body Weight

**Figure 4:**
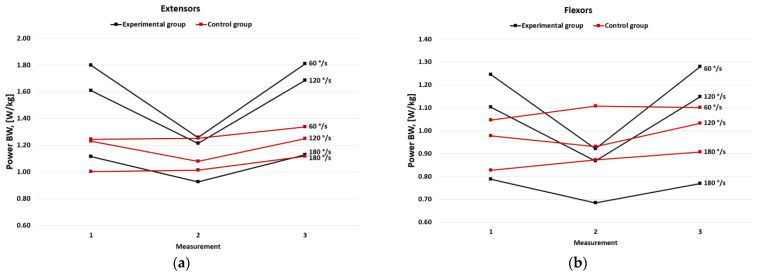
Mean power per body weight in the experimental and the control group for (**a**) extensors and (**b**) flexors.

In the experimental group, MP per BW significantly decreased in measurement no. 2 and then returned to the baseline in measurement no. 3 (*p* < 0.05). No differences were shown in the control group (Figure 4)

### 3.4. Time to Peak Torque

**Figure 5:**
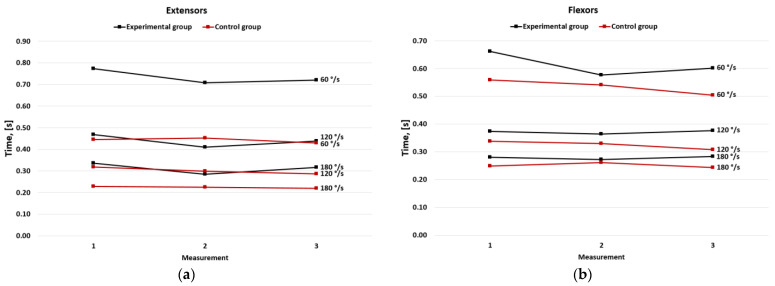
Mean of time to peak torque in the experimental and the control group for (**a**) extensors and (**b**) flexors.

In the experimental group, TPT in the extensors lowered significantly between the first and the second measurement (*p* < 0.05) and remained at this level at the third measurement in all three speeds (Figure 4a). Similar changes were observed in the TPT in the flexors only in the measurement in 60 °/s (Figure 4b). In the control group, no significant changes were observed in all speeds (Figure 5).

### 3.5. Flexors–Extensors Ratio

In general, flexors to extensors ratio values calculated for all measured parameters showed no significant differences among all three measurements. There were some significant differences observed in the post hoc Dunn–Bonferroni test in the experimental group for time to PT ratio in 180°/s between measurements no. 1 and 2 and in the control group for PT ratio in 60 °/s between measurements no. 1 and 2, WR ratio in 60 °/s between measurements no. 2 and 3, WR ratio in 180 °/s between measurement no. 1 and 2, and MP ratio in 180 °/s between measurement no. 1 and 2. Nevertheless, all parameters showed no differences in the flexors–extensors ratio between the first and the third measurements.

All detailed values of the calculated parameters are in Appendix A, including measurements in 60 °/s (Table A1), 120 °/s (Table A2), and 180 °/s (Table A3).

## 4. Discussion

The objective of this study was to assess the effectiveness of flossing applied during a warm-up in improving the strength and time parameters of the knee flexors and extensors. It was shown that floss band application during exercises does not improve generated peak torque, power, or work immediately after the warm-up. Furthermore, muscle strength parameters were significantly lower during exercising with a floss band than without it. A slight increase in muscle strength in the control group suggests, that the same set of exercises without flossing is even more effective in improving muscle strength parameters than with floss band application. There were some changes observed in the time to peak-torque and flexors–extensors ratio, but they seem to be clinically irrelevant.

Among the current literature, few studies on floss bands can be directly compared with the present investigation. Most published works have analysed Starett’s and Cordoza’s [8] original assumptions and examined the effectiveness of tissue flossing in improving range of motion, overall joint performance, and tissue flexibility [8,14]. Studies have predominantly been carried out on athletes and in the setting of post-injury rehabilitation for sports. Most of the protocols involved several repeated flossing sessions combined with sports practice sessions, and the outcomes are inconsistent [1,12,13,26,37]. Such outcomes are also reported after a single session of floss band intervention [13,26,27,28,35,38,39].

Chang et al. [38] reported a significant increase in quadriceps peak torque per BW and a decrease in hamstrings peak torque per BW immediately after single floss band application. Intervention with a floss band was at some points different from the intervention in the present study, as it included walking knee lift, side squat, and lunge and lasted 3 min. In the present study, participants performed high-intensity exercises in an open kinetic chain (isokinetic measurement), which also lasted about 3 min, but the intensity and biomechanics of muscle contraction were different. Although Chang et al. [38] did not calculate a flexors–extensors ratio, it seems that the direction of this ratio changed in their study from above 1 before, to less than 1 immediately after flossing and coming to baseline in 20 min after exercises. Such a result was not confirmed in the present study and by other researchers, as the flexors–extensors ratio is expected to be close to 0.60 when tested with a speed of 60 °/s [22]. It is worth noting that in the cited paper, the torque generated by the hamstring muscles before and 20 min after the intervention remained unchanged. In contrast, the quadriceps torque increased. In addition, in both cases, the hamstring torque is almost twice as high as that achieved for the quadriceps muscle. This makes the presented results significantly different from those recorded in our work.

Vogrin et al. [34] assessed nineteen recreational athletes in three different conditions (high floss band pressure, low floss band pressure, and control). They reported small to medium benefits associated with flossing application compared to controls regarding maximum voluntary contraction in isometric conditions for knee extensors (421.37 Nm before and 445.94 Nm after flossing with low pressure; 418.47 Nm before and 429.16 Nm after flossing with high pressure) and unclear to small benefits for flexors (215.82 Nm before and 220.26 Nm after flossing; 203.33 Nm before and 211.07 Nm after flossing with high pressure). A slight increase in the muscle torque obtained for flexors seems to be comparable with the results presented above, while those for extensors seem to be better than those achieved in the present study, although they did not refer to body weight. Konrad et al. [27] reported a slight increase in maximal isometric knee extension (from 293.10 Nm to 309.56 Nm), while Kaneda et al. [35] confirmed these results for isometric knee flexion (63.8 to 66.3% of body weight). However, Konrad et al. [27] reported no group effect. Both studies were performed on relatively small study groups (16 and 17 recreational male athletes, respectively) and the increase in muscle strength was comparable with those reported for flexors peak torque per body weight in the present study, although we noticed non-significant changes. Kaneda et al. [35] measured also maximal eccentric knee flexion and extension torque. They reported an average increase of almost 30% BW for knee extension and 10% BW for knee flexion after flossing application [35]. The methodology of this study was in some points different from the above-cited studies: 70 athletes were assessed and divided into two groups, included also female participants. Additionally, concentric peak torque in isokinetic conditions was measured instead of isometric or isokinetic eccentric peak torque. Therefore, direct comparison between the results of these studies is limited.

The present study was based on a large study population of recreational athletes. It is similar in this respect to the publications of Driller et al., who assessed the effect of tissue flossing on the ankle joint and the ability to jump and run [13,26,28]. The results were positive, as the authors stated that flossing can contribute to the prevention of injuries by improving joint mobility and that it can be used during warm-up among both recreational athletes and elite rugby union athletes. The first of these studies showed a significant improvement in height (from 23 to 27 cm) and velocity (from 1.88 to 2.03 m/s) in a single-leg vertical jump test immediately following the application of a flossing band, possibly indicating improved muscle strength and power [13]. These findings, however, were not confirmed in another study, where the persistence of the effects of flossing was assessed using a counter-movement jump (CMJ) test [28]. There were no significant time and intervention interaction effects in the CMJ test results for either the study or control group, but there were small benefits associated with flossing 30 min after application. In addition, there were no differences between the study and control groups in the CMJ test results in a similar study involving elite rugby union athletes [26].

The positive influence of warm-up visible in the control group in the present study was confirmed by Paravlic et al. [40] in their study assessing tensiomyography (TMG) parameters and the CMJ test. At the same time, they reported negative alterations in all TMG parameters and reduced CMJ test results immediately after flossing, which corresponds with the results of the present study. This can suggest that ischemic preconditioning can reduce athletic performance [41,42], which can increase the risk of an injury, especially during competition.

The most noticeable finding in the present study was a marked decrease in the values of the strength parameters investigated in the isokinetic study when the floss band was applied. Blacker et al. [43] showed that knee isokinetic measurements taken within one day have high reproducibility. The results obtained by a control group in the present study, confirm this. Therefore, the effect we observed could be due to a sense of discomfort or pain caused by pressure on the muscles from the tight band, direct contact of the taut rubber with the skin or an additional reduction in comfort as the subjects sat in the chair of the dynamometer with the floss band. While such sensations are subjective experiences, they may affect one’s motivation to perform a task at maximum effort. This observation should be considered in future studies on this topic.

A decrease in muscle strength with flossing is considered a sign that this technique should not be used in exercises that require muscle strength and power. In this regard, the work generated by muscles after a floss band was applied can be compared to that after static stretching, which has an undesirable effect on muscles during warm-up, reducing the generated muscle strength by 3% to 9%. Different authors have reported that dynamic stretching can improve muscle strength by as much as 9% or cause a slight decrease by 4%, and some authors have found no effect on the force generated [44,45]. The findings of the present study indicate that tissue flossing ranks between static and dynamic stretching concerning its effect on strength parameters. The application of a floss band resulted in decreases in most parameters tested, but on the third measurement, after the floss band had been removed, the parameters returned to baseline or near-baseline values. On one hand, this may be a sign that the effects of flossing have a short duration or do not influence muscle strength directly after use. On the other hand, a slight increase in strength parameters in the control group suggests, that the influence of tissue flossing on muscles is not positive when analysed directly after exercises with a floss band. Therefore, there is no rationale for using soft tissue flossing during warm-up to improve muscle strength parameters before the main exercise session or competition.

Although an innovative approach to assessing the effectiveness of flossing was applied, the present study has some limitations. The floss-band stretch force was not quantified [34,46] and the blood flow restriction is also not quantifiable [47]. Possible differences in band application were minimized by involving only one, highly experienced physiotherapist in this task. The study protocol also did not include fatigue parameters, especially from high-speed tests. Calculating the fatigue ratio for different strength and time variables could give additional information about the influence of tissue flossing on muscle physiology. This study, due to its design, was not blinded to participants or investigators.

In future studies, it would be worthful to analyse, how tissue flossing application applied during warm-up influences results achieved in the main session or during competition. It is warranted to consider different pressure levels, intensity, and duration of warm-up exercises, as well as the type of sports activities to draw reliable conclusions.

## 5. Conclusions

Based on the above-described results and their comparison to the literature, it can be concluded that a single session of muscle flossing does not improve muscle strength or power and may even decrease maximum muscle strength. It is therefore not recommended for use during warm-up. Additional research is warranted to determine the effects of flossing on other muscle-related indices, such as muscle excitability, endurance or contraction velocity.

## Figures and Tables

**Table 1:** Characteristics of the participants (mean ± SD).

Group	Gender	Age (Years)	Body Mass (kg)	Body Height (cm)	Body Mass Index BMI (kg/m^2^)
Experimental group (*n* = 36)	21 females 15 males	21.0 ± 2.1	68.8 ± 12.9	171.8 ± 10.9	23.2 ± 2.9
Control group (*n* = 30)	24 females 6 males	21.8 ± 2.2	65.9 ± 12.9	170.2 ± 8.2	22.6 ± 2.9

## Data Availability

The measurement data used to support the findings of this study are available from the corresponding author upon request.

## Appendix A

**Table A1:** Results of the measurement in the 60 °/s test for the experimental and the control group, where: s—means small, m—medium and l—large effect size calculated by Cohen’s d.

Group	Parameter	Measurement 1	*p*-Value 1–2	Measurement 2	*p*-Value 2–3	Measurement 3	*p*-Value 1–3
Experimental group	Ext peak torque BW [Nm/kg]	Me 1.75 IQR 1.49–2.06	<0.001 m	Me 1.51 IQR 1.28–1.81	0.017 s	Me 1.70 IQR 1.39–2.03	0.949 s
	Flx peak torque BW [Nm/kg]	Me 1.09 IQR 0.89–1.25	0.007 s	Me 0.97 IQR 0.88–1.12	0.002 m	Me 1.12 IQR 0.98–1.30	>0.999 s
	Peak torque flx-ext ratio	Me 0.60 IQR 0.55–0.68	0.065 s	Me 0.65 IQR 0.61–0.73	>0.999 s	Me 0.66 IQR 0.56–0.74	0.335 s
	Ext work BW [Nm/kg]	Me 2.09 IQR 1.82–2.43	<0.001 s	Me 1.76 IQR 1.43–2.23	<0.001 s	Me 2.02 IQR 1.66–2.58	>0.999 s
	Flx work BW [Nm/kg]	Me 1.43 IQR 1.19–1.69	<0.001 s	Me 1.16 IQR 0.98–1.42	0.001 s	Me 1.40 IQR 1.21–1.69	>0.999 s
	Work flx-ext ratio	Me 0.68 IQR 0.60–0.76	>0.999 s	Me 0.71 IQR 0.62–0.80	>0.999 s	Me 0.67 IQR 0.58–0.78	>0.999 s
	Ext power BW [W/kg]	Me 1.08 IQR 0.95–1.32	0.010 m	Me 0.86 IQR 0.74–1.16	0.007 m	Me 1.08 IQR 0.90–1.39	>0.999 s
	Flx power BW [W/kg]	Me 0.79 IQR 0.64–0.91	0.004 m	Me 0.67 IQR 0.62–0.78	0.003 s	Me 0.77 IQR 0.66–0.91	>0.999 s
	Power flx-ext ratio	Me 0.70 IQR 0.63–0.82	>0.999 s	Me 0.76 IQR 0.65–0.85	>0.999 s	Me 0.72 IQR 0.59–0.80	>0.999 s
	Ext time to peak torque [s]	Me 0.77 IQR 0.615–0.895	0.047 s	Me 0.69 IQR 0.54–0.81	>0.999 s	Me 0.735 IQR 0.595–0.87	0.422 s
	Flx time to peak torque [s]	Me 0.605 IQR 0.50–0.81	0.231 s	Me 0.545 IQR 0.465–0.65	>0.999 s	Me 0.585 IQR 0.49–0.64	0.867 s
	Time to PT flx-ext ratio	Me 0.86 IQR 0.72–1.00	>0.999 s	Me 0.87 IQR 0.68–1.07	>0.999 s	Me 0.79 IQR 0.69–0.99	>0.999 s
Control group	Ext peak torque BW [Nm/kg]	Me 1.63 IQR 1.13–2.26	0.736 s	Me 1.82 IQR 1.10–2.15	0.244 s	Me 1.90 IQR 1.40–2.24	>0.999 s
	Flx peak torque BW [Nm/kg]	Me 1.13 IQR 0.83–1.43	0.413 s	Me 1.21 IQR 1.01–1.34	>0.999 s	Me 1.25 IQR 0.98–1.46	>0.999 s
	Peak torque flx-ext ratio	Me 0.68 IQR 0.56–0.78	0.024 l	Me 0.68 IQR 0.62–0.81	0.212 s	Me 0.69 IQR 0.54–0.78	>0.999 s
	Ext work BW [Nm/kg]	Me 1.30 IQR 0.80–1.61	>0.999 s	Me 1.27 IQR 1.01–1.64	>0.999 s	Me 1.45 IQR 0.98–1.70	>0.999 s
	Flx work BW [Nm/kg]	Me 1.24 IQR 0.69–1.49	0.212 s	Me 1.21 IQR 0.86–1.43	0.590 s	Me 1.22 IQR 0.86–1.40	>0.999 s
	Work flx-ext ratio	Me 0.90 IQR 0.66–0.98	0.364 s	Me 0.91 IQR 0.81–1.13	0.020 s	Me 0.82 IQR 0.68–1.00	0.735 s
	Ext power BW [W/kg]	Me 0.96 IQR 0.62–1.34	>0.999 s	Me 1.00 IQR 0.62–1.34	0.051 s	Me 1.06 IQR 0.79–1.38	0.117 s
	Flx power BW [W/kg]	Me 0.83 IQR 0.59–1.05	>0.999 s	Me 0.86 IQR 0.77–0.99	>0.999 s	Me 0.95 IQR 0.73–1.10	0.504 s
	Power flx-ext ratio	Me 0.91 IQR 0.67–1.00	0.051 s	Me 0.89 IQR 0.63–1.16	0.071 s	Me 0.83 IQR 0.68–1.00	>0.999 s
	Ext time to peak torque [s]	Me 0.42 IQR 0.34–0.50	>0.999 s	Me 0.43 IQR 0.35–0.56	>0.999 s	Me 0.445 IQR 0.36–0.49	>0.999 s
	Flx time to peak torque [s]	Me 0.54 IQR 0.48–0.67	>0.999 s	Me 0.55 IQR 0.49–0.61	0.107 s	Me 0.50 IQR 0.43–0.56	0.302 s
	Time to PT flx-ext ratio	Me 1.35 IQR 1.02–1.59	0.198 s	Me 1.21 IQR 0.88–1.50	>0.999 s	Me 1.16 IQR 0.94–1.46	0.567 s

Ext—extersors; Flx—flexors; PT—peak torque; BW—body weight; Me—median; IQR—inter-quartile range; *p*-values for post hoc Dunn–Bonferroni test.

**Table A2:** Results of the measurement in the 120 °/s test for the experimental and the control group, where: s—means small, m—medium and l—large effect size calculated by Cohen’s d.

	Parameter	Measurement 1	*p*-Value 1–2	Measurement 2	*p*-Value 2–3	Measurement 3	*p*-Value 1–3
Experimental group	Ext peak torque BW [Nm/kg]	Me 1.33 IQR 1.09–1.52	<0.001 m	Me 1.01 IQR 0.77–1.19	<0.001 m	Me 1.28 IQR 1.10–1.61	>0.999 s
	Flx peak torque BW [Nm/kg]	Me 0.85 IQR 0.77–0.95	<0.001 l	Me 0.71 IQR 0.56–0.75	<0.001 l	Me 0.91 IQR 0.79–1.04	>0.999 s
	Peak torque flx-ext ratio	Me 0.67 IQR 0.56–0.75	>0.999 s	Me 0.70 IQR 0.60–0.83	>0.999 s	Me 0.67 IQR 0.59–0.77	>0.999 s
	Ext work BW [Nm/kg]	Me 1.57 IQR 1.37–2.00	<0.001 l	Me 1.16 IQR 0.98–1.42	<0.001 l	Me 1.63 IQR 1.40–1.99	>0.999 s
	Flx work BW [Nm/kg]	Me 1.12 IQR 0.92–1.33	<0.001 l	Me 0.83 IQR 0.69–1.00	<0.001 l	Me 1.13 IQR 0.98–1.34	0.867 s
	Work flx-ext ratio	Me 0.69 IQR 0.60–0.80	>0.999 s	Me 0.71 IQR 0.64–0.84	>0.999 s	Me 0.66 IQR 0.61–0.81	>0.999 s
	Ext power BW [W/kg]	Me 1.45 IQR 1.35–1.91	<0.001 m	Me 1.18 IQR 0.90–1.46	<0.001 l	Me 1.56 IQR 1.32–2.06	>0.999 s
	Flx power BW [W/kg]	Me 1.13 IQR 0.94–1.34	0.002 l	Me 0.84 IQR 0.70–0.99	<0.001 l	Me 1.18 IQR 0.97–1.33	>0.999 s
	Power flx-ext ratio	Me 0.72 IQR 0.63–0.82	>0.999 s	Me 0.73 IQR 0.67–0.88	0.472 s	Me 0.71 IQR 0.62–0.81	>0.999 s
	Ext time to peak torque [s]	Me 0.46 IQR 0.415–0.525	0.003 m	Me 0.405 IQR 0.34–0.46	>0.999 s	Me 0.43 IQR 0.40–0.475	0.020 s
	Flx time to peak torque [s]	Me 0.385 IQR 0.31–0.425	0.262 s	Me 0.37 IQR 0.31–0.40	0.789 s	Me 0.37 IQR 0.335–0.425	>0.999 s
	Time to PT flx-ext ratio	Me 0.80 IQR 0.68–0.93	0.178 s	Me 0.85 IQR 0.76–1.03	>0.999 s	Me 0.85 IQR 0.76–0.99	0.297 s
Control group	Ext peak torque BW [Nm/kg]	Me 1.07 IQR 0.69–1.37	>0.999 s	Me 1.13 IQR 0.83–1.37	0.881 s	Me 1.15 IQR 0.89–1.64	0.263 s
	Flx peak torque BW [Nm/kg]	Me 0.79 IQR 0.60–1.10	>0.999 s	Me 0.89 IQR 0.66–1.07	>0.999 s	Me 0.95 IQR 0.66–1.19	>0.999 s
	Peak torque flx-ext ratio	Me 0.78 IQR 0.59–0.96	>0.999 s	Me 0.80 IQR 0.64–1.00	0.054 s	Me 0.75 IQR 0.57–0.87	0.147 s
	Ext work BW [Nm/kg]	Me 0.79 IQR 0.63–1.25	>0.999 s	Me 0.86 IQR 0.69–1.28	>0.999 s	Me 0.95 IQR 0.98–1.55	>0.999 s
	Flx work BW [Nm/kg]	Me 0.79 IQR 0.54–0.95	>0.999 s	Me 0.83 IQR 0.51–1.10	>0.999 s	Me 0.83 IQR 0.48–1.10	>0.999 s
	Work flx-ext ratio	Me 0.94 IQR 0.65–1.07	0.345 s	Me 0.93 IQR 0.74–1.16	0.054 s	Me 0.82 IQR 0.70–1.05	>0.999 s
	Ext power BW [W/kg]	Me 1.14 IQR 0.88–1.56	>0.999 s	Me 1.23 IQR 0.88–1.58	>0.999 s	Me 1.27 IQR 0.95–1.91	0.567 s
	Flx power BW [W/kg]	Me 1.01 IQR 0.79–1.43	>0.999 s	Me 1.12 IQR 0.79–1.36	>0.999 s	Me 1.20 IQR 0.79–1.34	>0.999 s
	Power flx-ext ratio	Me 0.94 IQR 0.66–1.08	0.263 s	Me 0.95 IQR 0.79–1.21	0.077 s	Me 0.85 IQR 0.73–1.06	>0.999 s
	Ext time to peak torque [s]	Me 0.31 IQR 0.26–0.36	0.637 s	Me 0.30 IQR 0.24–0.34	>0.999 s	Me 0.28 IQR 0.25–0.32	0.567 s
	Flx time to peak torque [s]	Me 0.32 IQR 0.28–0.37	>0.999 s	Me 0.33 IQR 0.27–0.38	0.147 s	Me 0.28 IQR 0.25–0.35	0.504 s
	Time to PT flx-ext ratio	Me 1.02 IQR 0.76–1.36	0.302 s	Me 1.13 IQR 0.95–1.26	0.263 s	Me 1.10 IQR 0.88–1.21	>0.999 s

Ext—extersors; Flx—flexors; PT—peak torque; BW—body weight; Me—median; IQR—inter-quartile range; *p*-values for post hoc Dunn–Bonferroni test.

**Table A3:** Results of measurement in the 180 °/s test for the experimental and control groups, where: s—means small, m—medium and l—large effect size calculated by Cohen’s d.

	Parameter	Measurement 1	*p*-Value 1–2	Measurement 2	*p*-Value 2–3	Measurement 3	*p*-Value 1–3
Experimental group	Ext peak torque BW [Nm/kg]	Me 1.06 IQR 0.88–1.25	<0.001 s	Me 0.74 IQR 0.57–0.92	<0.001 s	Me 1.07 IQR 0.85–1.25	>0.999 s
	Flx peak torque BW [Nm/kg]	Me 0.74 IQR 0.65–0.82	<0.001 s	Me 0.59 IQR 0.47–0.66	<0.001 s	Me 0.74 IQR 0.63–0.88	>0.999 s
	Peak torque flx-ext ratio	Me 0.71 IQR 0.58–0.79	0.585 s	Me 0.71 IQR 0.64–0.80	>0.999 s	Me 0.71 IQR 0.57–0.88	0.526 s
	Ext work BW [Nm/kg]	Me 1.24 IQR 1.01–1.53	<0.001 s	Me 0.83 IQR 0.63–1.06	<0.001 s	Me 1.21 IQR 1.01–1.52	>0.999 s
	Flx work BW [Nm/kg]	Me 0.86 IQR 0.77–1.01	<0.001 s	Me 0.68 IQR 0.51–0.79	<0.001 s	Me 0.91 IQR 0.76–1.06	>0.999 s
	Work flx-ext ratio	Me 0.70 IQR 0.61–0.81	>0.999 s	Me 0.77 IQR 0.62–0.86	>0.999 s	Me 0.73 IQR 0.60–0.86	>0.999 s
	Ext power BW [W/kg]	Me 1.69 IQR 1.39–2.11	<0.001 s	Me 1.19 IQR 0.87–1.52	<0.001 s	Me 1.77 IQR 1.37–2.25	>0.999 s
	Flx power BW [W/kg]	Me 1.26 IQR 1.08–1.44	<0.001 s	Me 0.97 IQR 0.71–1.12	<0.001 s	Me 1.27 IQR 1.04–1.59	>0.999 s
	Power flx-ext ratio	Me 0.72 IQR 0.60–0.81	>0.999 s	Me 0.77 IQR 0.62–0.89	>0.999 s	Me 0.72 IQR 0.60–0.89	>0.999 s
	Ext time to peak torque [s]	Me 0.335 IQR 0.30–0.36	<0.001 s	Me 0.28 IQR 0.255–0.32	0.789 s	Me 0.31 IQR 0.285–0.33	0.020 s
	Flx time to peak torque [s]	Me 0.28 IQR 0.255–0.31	0.648 s	Me 0.27 IQR 0.235–0.31	>0.999 s	Me 0.27 IQR 0.245–0.30	>0.999 s
	Time to PT flx-ext ratio	Me 0.82 IQR 0.73–0.95	0.029 m	Me 0.92 IQR 0.80–1.10	0.789 s	Me 0.87 IQR 0.75–1.00	0.422 s
Control group	Ext peak torque BW [Nm/kg]	Me 0.83 IQR 0.63–1.07	>0.999 s	Me 0.72 IQR 0.57–0.98	0.125 s	Me 0.83 IQR 0.66–1.13	0.567 s
	Flx peak torque BW [Nm/kg]	Me 0.63 IQR 0.48–0.89	>0.999 s	Me 0.69 IQR 0.36–0.83	>0.999 s	Me 0.74 IQR 0.45–0.83	>0.999 s
	Peak torque flx-ext ratio	Me 0.80 IQR 0.68–1.00	0.446 s	Me 0.77 IQR 0.67–1.00	0.567 s	Me 0.77 IQR 0.64–1.00	>0.999 s
	Ext work BW [Nm/kg]	Me 0.65 IQR 0.42–0.89	0.712 s	Me 0.57 IQR 0.42–0.75	0.504 s	Me 0.63 IQR 0.51–0.92	>0.999 s
	Flx work BW [Nm/kg]	Me 0.53 IQR 0.39–0.72	>0.999 s	Me 0.57 IQR 0.27–0.77	>0.999 s	Me 0.59 IQR 0.33–0.80	>0.999 s
	Work flx-ext ratio	Me 0.83 IQR 0.68–1.00	0.031 s	Me 0.91 IQR 0.70–1.11	0.147 s	Me 0.78 IQR 0.61–1.00	>0.999 s
	Ext power BW [W/kg]	Me 1.22 IQR 0.81–1.63	>0.999 s	Me 1.08 IQR 0.77–1.30	0.198 s	Me 1.22 IQR 0.88–1.74	0.567 s
	Flx power BW [W/kg]	Me 0.96 IQR 0.68–1.27	>0.999 s	Me 0.95 IQR 0.53–1.34	>0.999 s	Me 1.06 IQR 0.68–1.43	>0.999 s
	Power flx-ext ratio	Me 0.84 IQR 0.67–0.96	0.012 s	Me 0.92 IQR 0.71–1.15	0.446 s	Me 0.81 IQR 0.65–1.03	0.446 s
	Ext time to peak torque [s]	Me 0.23 IQR 0.19–0.27	>0.999 s	Me 0.22 IQR 0.18–0.26	>0.999 s	Me 0.23 IQR 0.17–0.25	>0.999 s
	Flx time to peak torque [s]	Me 0.225 IQR 0.20–0.28	>0.999 s	Me 0.25 IQR 0.21–0.28	0.125 s	Me 0.22 IQR 0.20–0.26	0.263 s
	Time to PT flx-ext ratio	Me 1.05 IQR 0.95–1.22	0.881 s	Me 1.13 IQR 0.89–1.50	0.393 s	Me 0.98 IQR 0.83–1.40	>0.999 s

Ext—extersors; Flx—flexors; PT—peak torque; BW—body weight; Me—median; IQR—inter-quartile range; *p*-values for post hoc Dunn–Bonferroni test.
